# When Hearts and Minds Collide: A Case Report of Left Ventricle Noncompaction Syndrome as a Precursor to Acute Ischemic Stroke in a 15-Year-Old Along With a Literature Review

**DOI:** 10.7759/cureus.54168

**Published:** 2024-02-14

**Authors:** Yousif Alkhafaji, Omar Farooq Al-Nahhas, Khaled Alaboud Alkheder, Jamal A Alkoteesh

**Affiliations:** 1 Emergency Medicine, Tawam Hospital, Al Ain, ARE; 2 Interventional Radiology, Tawam Hospital, Al Ain, ARE

**Keywords:** left ventricle non-compaction, internal carotid artery (ica), middle cerebral artery (mca), : ischemic stroke, pediatric stroke, mechanical thrombectomy in pediatrics, weakness, stroke, cerebral vascular accident

## Abstract

Pediatric stroke, though uncommon, is often underdiagnosed due to subtle symptoms and delayed recognition. Cardiac diseases, accounting for up to 33% of pediatric ischemic strokes, play a significant role. This case report explores the rare occurrence of ischemic stroke in a 15-year-old boy with left ventricular non-compaction syndrome (LVNC). It underscores the complexity of managing pediatric ischemic stroke, particularly in the context of LVNC, emphasizing the challenges in timely diagnosis and management.

## Introduction

It is rare for children to experience a stroke or cerebral vascular accident (CVA). The incidence of combined ischemic and hemorrhagic pediatric stroke ranges from 1.2 to 13 cases per 100,000 children under 18 years of age [1]. However, pediatric stroke may be more common than we think because it is often misdiagnosed or undiagnosed. This could be due to a variety of factors, including a clinician's low level of suspicion and patients who exhibit subtle symptoms that resemble other diseases. As a result, the diagnosis of a stroke may be delayed. A report revealed that 19 out of 45 children with a stroke were not correctly diagnosed until 15 hours to 3 months after their initial presentation [2]. Another study showed that seeking medical attention was delayed up to 28 hours from the onset of symptoms and 7.2 hours on average after presentation before any brain imaging was performed [3].

In childhood, cardiac disease is the leading factor behind stroke, responsible for up to 33% of all cases of acute ischemic stroke among all other causes of stroke [4].

## Case presentation

A 15-year-old male of Middle Eastern race presented to the emergency department accompanied by his parents. They reported that their son went to sleep at 1500 hrs, and they later found him at 1700 hrs in what they described as active tonic-clonic movements with frothing around the mouth, likely suggesting a seizure. He arrived at the emergency department at 1845 hrs in what appeared to be a state of post-ictal confusion and was found to have slurring of speech, right-sided facial weakness, and right-sided upper and lower limb power of 1/5. He was able to close his eyes normally, could elevate both eyebrows, and his plantar reflex was upgoing. The calculated National Institution of Health Stroke Scale (NIHSS) score was 21 on arrival.

A stroke code was activated, and the patient underwent a plain and contrast-enhanced CT brain and angiography. The plain CT brain scan showed a left middle cerebral artery (MCA) dense sign but no other abnormalities were detected. The CT brain angiography showed attenuated left intracranial internal carotid artery (ICA). Filling defects were seen in relation to the left distal/terminal ICA and the M1 segment of the left MCA. Flow seen within the insular branches of the left MCA and cortical branches of the left MCA are attenuated. His CT brain perfusion images revealed reduced cerebral blood flow and cerebral blood volume in the head of the left caudate nucleus and lentiform nucleus suggestive of infarction. The left MCA territory shows a large area of prolonged time to peak suggestive of ischemia.

As the patient was younger than 18, a discussion between the emergency physician, neurologist, and pediatrician with the parents agreed that intravenous thrombolysis has not been Food and Drug Administration (FDA)-approved for use in the pediatric age, and the child might have a better outcome with mechanical thrombectomy. Given that, interventional radiology was consulted, and the patient was taken for a mechanical intra-arterial thrombectomy of the left internal carotid and left middle cerebral artery occlusion.

Technique and findings

**Figure 1:**
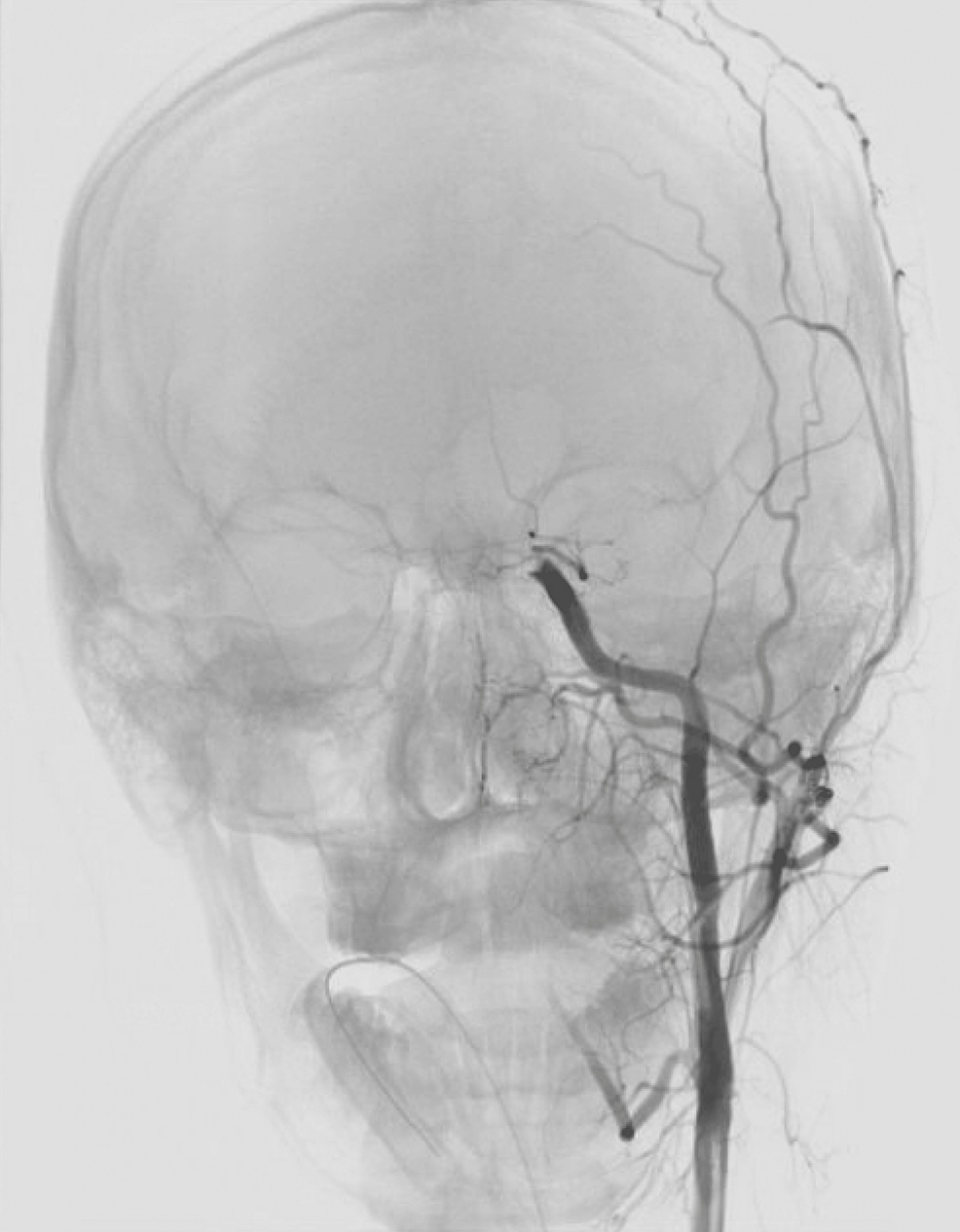
Angiography demonstrating left ICA T-occlusion and the M1 segment of the left MCA ICA: internal carotid artery; MCA: middle cerebral artery

The procedure was performed under general anesthesia with the patient in the supine position and undertaking the usual sterile technique. A 6 French NeuroMax sheath (Penumbra, CA, USA) was retrogradely placed within the right common femoral artery. A constant saline drip was maintained through the sheath for the duration of the procedure. A 5 French diagnostic catheter was used coaxially through a 6 French NeuroMax guiding catheter to selectively catheterize the left common carotid artery using regular fluoroscopy and road mapping. Angiographic control runs were performed through the 5 French diagnostic catheter and demonstrated occlusion of the left ICA T-occlusion and M1 segment of the left middle cerebral artery (Figure 1).

**Figure 2:**
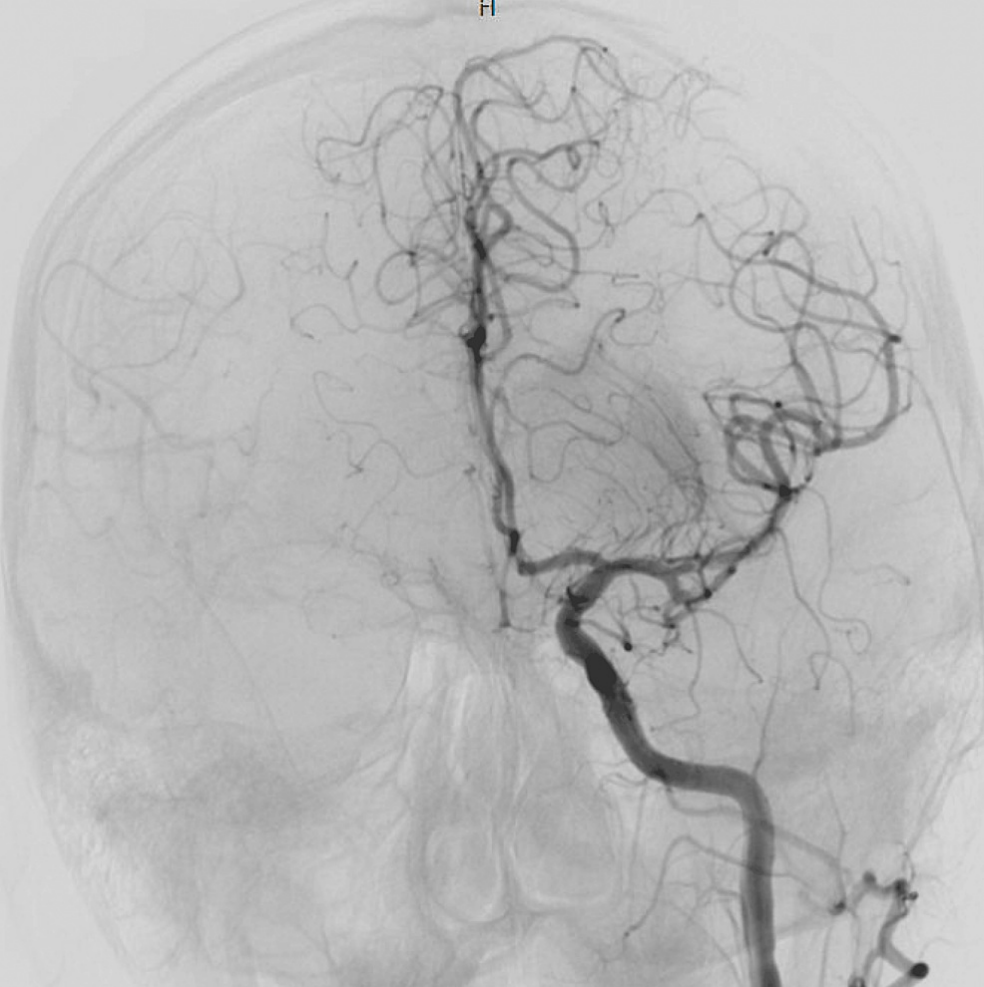
Angiography demonstrating complete recanalization of the left ICA/MCA consistent with TICI 3 recanalization TICI: thrombolysis in cerebral infarction; ICA: internal carotid artery; MCA: middle cerebral artery

We then proceeded with the mechanical aspiration thrombectomy procedure. A Rebar™ microcatheter (Medtronic, Dublin, Ireland) coaxially placed within a 6 French ACE 68 reperfusion catheter was navigated into the left ICA nas mest MCA/ M1 with the aid of a Silver Speed microguide wire. Then, the reperfusion catheter was advanced over the microcatheter into the M1 segment of the LEFT middle cerebral artery. We applied continuous suction using the Penumbra aspiration device through the side port of the ACE 68 reperfusion catheter. The angiographic runs of the left internal carotid artery demonstrated complete recanalization of the left ICA/MCA consistent with thrombolysis in cerebral infarction (TICI) 3 recanalization (Figure 2).

At the end of the procedure, the guidewire and catheter were removed. The groin sheath was kept in place and removed the next day. The procedure was well-tolerated and there were no immediate complications.

Hospital course

The patient was subsequently transferred to the Stroke unit post-mechanical thrombectomy. A few hours after the thrombectomy, the patient’s clinical examination was as follows: awake and oriented to time place, and person, he was able to answer simple questions and follow simple one-step commands. He could name objects but not repeat them. He had expressive aphasia and left gaze preference; however, he was able to cross the midline to the right. He had no blinking to threat on the right side, as well as a right-sided facial weakness. His power was 4+/5 on the right upper and lower limbs and the calculated NIHSS score was 6.

Subsequently, 15 hours post-thrombectomy, the patient underwent MRI brain-stroke protocol and magnetic resonance angiography (MRA) which revealed an area of restricted diffusion and low signal in apparent diffusion coefficient (ADC) sequence in the left head of the caudate, external, and internal capsules as well as the lentiform nucleus, with mild mass effect upon the left anterior horn of the lateral ventricle corresponding to recent infarction. Focal left parietal lobe foci of petechial hemorrhage were noted, however, no major intracerebral bleed was seen in the susceptibility-weighted imaging (SWI) sequence. There was a resolution of the filling defect in the left ICA and MI segments of the left MCA. The right MCA and both anterior cerebral artery (ACA) and posterior circulation were intact.

On day 1 post-mechanical thrombectomy, his power was 5/5 in both upper and lower limbs bilaterally. His sensations were normal bilaterally, he could follow commands and speak fluently, but sometimes he spoke sentences unrelated to the topic discussed. His facial weakness has decreased; however, it was still present.

**Figure 3:**
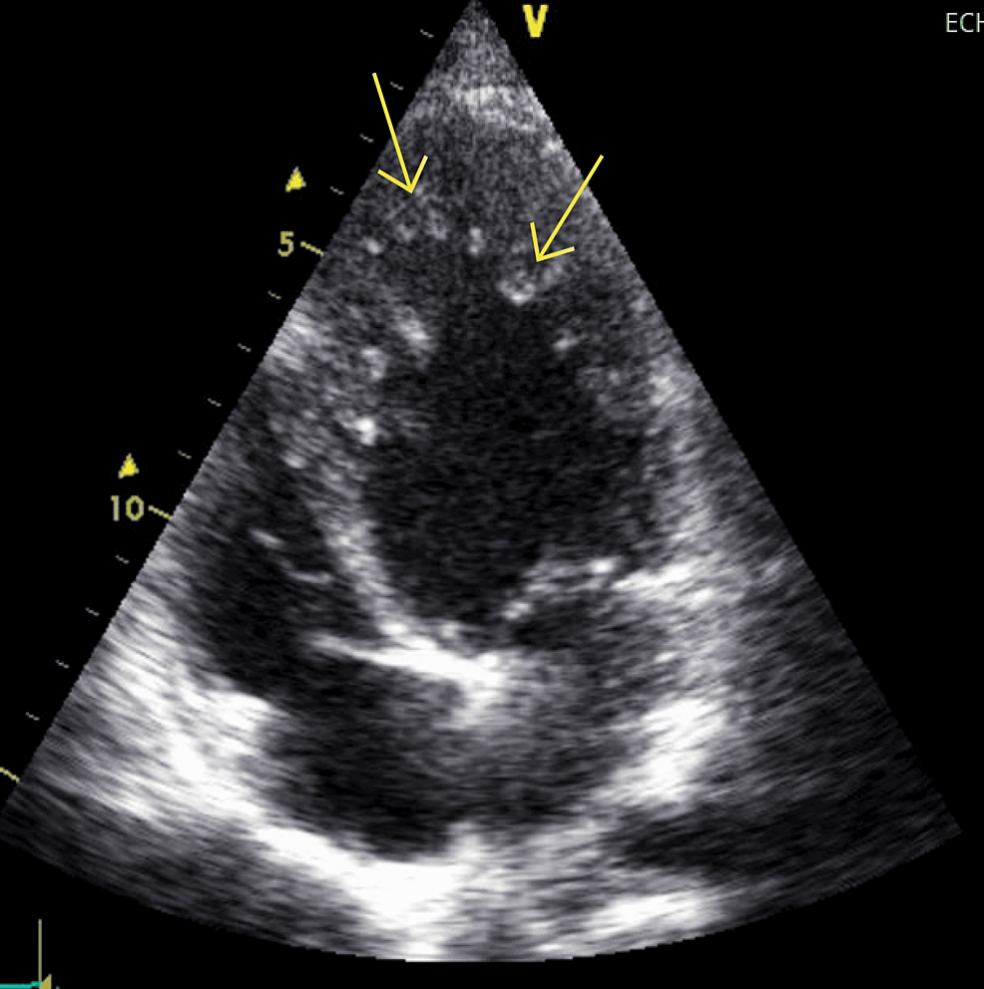
Trans-thoracic echocardiography Normal LV size with prominent trabeculations (arrows) in the LV cavity is consistent with noncompaction cardiomyopathy. LV: left ventricle

During his hospital course, he underwent transoesophageal echocardiography, which showed a normal-sized left ventricle (LV) with prominent trabeculations in the LV cavity, consistent with noncompaction cardiomyopathy: ejection fraction (EF) 50%(Simpson's), mild to moderate MR, mitral inflow (A tachycardia), and aneurysmal atrial septum. No spontaneous shunt was seen but he is known to have a patent foramen ovale (PFO)/atrial septal defect (ASD). No other abnormalities were detected (Figure 3).

The findings of non-compaction cardiomyopathy triggered a transfer to another tertiary care hospital with a pediatric cardiology service. He was started on aspirin following the stroke; however, no anticoagulation was initiated, as our cardiology team wanted to confirm the diagnosis with a cardiac MRI scan.

Before the patient’s transfer, a repeat brain MRI was obtained on his last day of hospitalization in our facility (10 days post-mechanical thrombectomy). The left caudate nucleus, basal ganglion, and part of the left MCA showed I2 shine suggesting a relatively old infarct. It showed that previously, there was distension of fluid moment in the rest of the brain parenchyma and brainstem. The cerebral hemisphere showed normal signal intensity and morphology. There was no evidence of an extra-axial collection, midline shift, or any bleeding.

His physical examination findings on day 10 were as follows: awake and oriented to place and person and able to answer simple questions and follow simple one-step commands or name objects; however, he showed some difficulty with reading. There was no right-left confusion, gaze preference, or visual field defect. He still had a right-sided facial weakness; however, he had a normal touch sensation on the face. His motor power on both upper and lower limbs was 5/5 bilaterally with normal sensations. The overall NIHSS score was 2.

**Figure 4:**
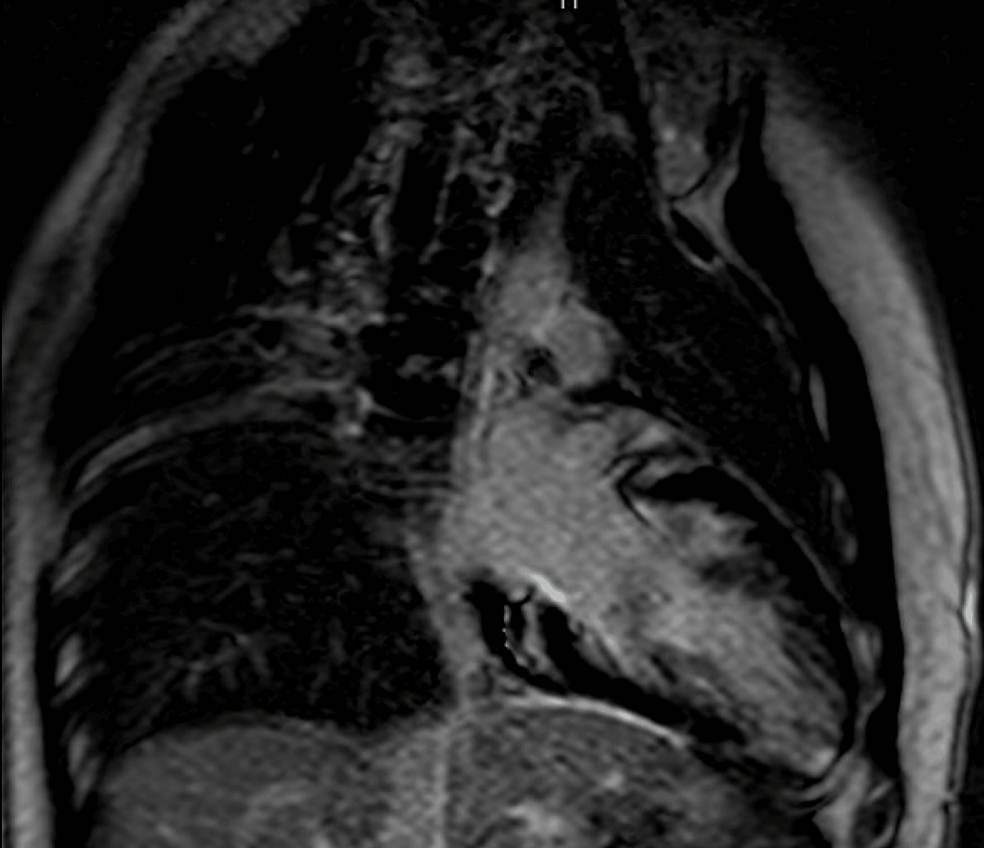
Cardiac MRI The ratio of non-compacted to compacted myocardium is more than 4:1, indicating LVNC cardiomyopathy, with non-compacted myocardium more prominent in the apex. LV: left ventricle; LVNC: left ventricle non-compaction

The patient was transferred to another facility, and during his stay at the pediatric cardiology service, he underwent a cardiac MRI scan that showed that the LV was dilated. LV regional wall motion demonstrated hypokinesia in all the mid- and apical segments with non-compacted myocardium more prominent in the apex. The ratio of non-compacted to compacted myocardium was more than 4:1, indicating LV noncompaction cardiomyopathy. The LV was significantly larger than the right ventricle (RV) and the apex was extremely thin showing severe hypokinesia. No other abnormalities were detected (Figure 4). Following the cardiac MRI scan, a thrombophilia screening was done, which revealed no abnormalities. Given his MRI findings, anticoagulation was initiated in the form of subcutaneous enoxaparin injections in a prophylactic weight-based dosage that was continued throughout his hospital stay.

On further exploration of the patient’s old health records and past medical history obtained from the parents, it was evident that the patient was diagnosed with non-compaction syndrome at the age of seven. He first presented, complaining of palpitations and physical effort intolerance for six months. An electrocardiogram showed junctional tachycardia and a long QT interval. Holter monitoring was done and showed the following: first-degree atrioventricular block, frequent monomorphic premature atrial/ventricular contractions occurring in about 2-3% of the beats, three recorded episodes of non-sustained SVT at about 130 bpm, one recorded episode of tachycardia at 171 bpm, but it's unclear whether it was sinus tachycardia or SVT with average QTc 493 ms. A stress exercise test was performed later on and revealed mild prolongation of QTc with tachycardia up to 0.46 sec. at peak exercise. Transthoracic echocardiography at that time showed normal LV dimensions with mild systolic dysfunction (EF 51%), mild apical LV myocardial non-compaction, and normal LV Z-scores. The patient was started on rate and rhythm control medication; however, he was not kept on any anticoagulation. A few years later, he suffered multiple frequent episodes of atrial tachycardia. He underwent radiofrequency ablation for his tachycardia focus; however, he still suffered tachycardic episodes.

Post-stroke, he was regularly followed up by the neurology and cardiology teams as an outpatient. He’s been doing well to date (three years post-stroke), with minimal residual facial deviation and otherwise no focal sensory or motor deficits.

## Discussion

Left ventricular non-compaction (LVNC) is a very rare congenital cardiomyopathy. It is a disease of endomyocardial trabeculations that increase in number and prominence. This cardiomyopathy carries a high risk of malignant arrhythmias, thromboembolic phenomenon, and left ventricular dysfunction. This disease also has other names like spongy myocardium, spongiform cardiomyopathy, hyper trabeculation, persisting myocardial sinusoids, or zaspopathy. It can carry associations with complex congenital heart defects or skeletal myopathy. It represents the arrest of the normal maturation process of the myocardium. This disease can present throughout life with progressive left ventricular systolic dysfunction.

LVNC is also known by various names such as spongy myocardium, spongiform cardiomyopathy, hyper trabeculation, persisting myocardial sinusoids, or zaspopathy. It represents an arrest of the normal maturation process of the myocardium, leading to endomyocardial trabeculations. These changes within the myocardium lead to a high risk of malignant arrhythmias, thromboembolic phenomenon, and left ventricular dysfunction [5]. Hirono et al. reported that the overall annual incidence rate of thromboembolic (TE) events in all patients with LVNC ranged from 0.08% to 0.27%[6].

In a study conducted by Stollberger and colleagues, they examined the occurrence of TE events in 62 individuals with LVNC and found that six individuals experienced TE events, of which 5 (9.7%) had systolic dysfunction. Based on their findings, they recommended that prophylaxis may be necessary for individuals with LVNC and systolic dysfunction [7]. Meanwhile, Pitta and colleagues suggested that anticoagulant or antiplatelet therapy should be considered for adults, especially those with heart failure (HF) or atrial fibrillation (AF) [8]. The incidence of TE events in children with LVNC remains unclear, but anticoagulant and/or antiplatelet therapy may be a viable option for those with reduced LV systolic function.

Management of ischemic stroke in children can be quite challenging, as further studies are needed to fully assess the safety of using intravenous thrombolytic therapy like tissue-type plasminogen activator (tPA) in patients under the age of 18. According to Lefond et al, the risk of symptomatic intracranial hemorrhage (SICH) after administering tPA to children is estimated to be 2.1%. They conclude that there is a probability of 98% that the risk of SICH is less than 15% and a 93% probability that it is less than 10%. These findings indicate that the overall risk of SICH after administering intravenous tPA to children with acute arterial ischemic stroke within 4.5 hours of symptom onset is low [9]. However, no clear guidelines exist on the inclusion and exclusion criteria for tPA, and most data suggest using adult criteria for exclusion. According to the American Heart Association (AHA), it is recommended that when intravenous tPA is being considered for children, an adult dose of 0.9 mg/kg should be used. However, this dosage may be conservative, as developmental differences in plasminogen levels may require a higher effective dosage for children [9].

When it comes to endovascular thrombectomy clear guidelines are lacking too. The published literature has documented over 35 cases of successful outcomes from recanalization therapy in pediatric arterial ischemic stroke (AIS). However, the exact number of patients who underwent thrombectomy treatment is still unclear. The AHA recommends limiting mechanical thrombectomy to children who meet the following criteria [9]: persistent disabling neurological deficits (eg, pediatric NIH Stroke Scale score ≥6 at the time of intervention or higher if DAWN trial criteria are being applied), radiographically confirmed cerebral large artery occlusion, larger children because of concerns about introducing catheters into the small groin and cerebral arteries and size-based limitations on contrast dye and radiation exposure, treatment decisions made in conjunction with neurologists with expertise in the treatment of children with stroke, and intervention performed by an endovascular surgeon with experience in both treating children and performing thrombectomy in adult stroke patients.

According to a retrospective analysis of registry data from the Save ChildS cohort study, 73 children with acute arterial ischemic stroke, aged between 0.7 to 18 years, underwent endovascular recanalization using aspiration or stent retriever devices. They concluded that the size of the stent retriever did not affect the rates of recanalization, complications, or overall outcomes. Additionally, there was no significant difference in these measures between the use of first-pass aspiration and other endovascular methods such as stent retrievers. The children experienced good neurological outcomes, and the rate of complications was low and did not vary between age groups.

## Conclusions

To sum up, there is not enough data to provide clear management guidelines for children with AIS. We suggest consulting with a pediatrician, pediatric neurologist, and interventional radiologist to decide and explain the associated risks and benefits, as well as the lack of clear management guidelines to the family. Additionally, further studies are needed to establish consensus on management. This case report contributes to the existing literature to help establish these guidelines.
